# Influence of revegetation on soil microbial community and its assembly process in the open-pit mining area of the Loess Plateau, China

**DOI:** 10.3389/fmicb.2022.992816

**Published:** 2022-08-25

**Authors:** Yuanyuan Chang, Fu Chen, Yanfeng Zhu, Yunnan You, Yanjun Cheng, Jing Ma

**Affiliations:** ^1^School of Public Policy and Management, China University of Mining and Technology, Xuzhou, China; ^2^School of Public Administration, Hohai University, Nanjing, China; ^3^School of Environment and Spatial Informatics, China University of Mining and Technology, Xuzhou, China; ^4^Engineering Research Center of Ministry of Education for Mine Ecological Restoration, Xuzhou, China

**Keywords:** vegetation restoration, microbial community, ecological network, assembly process, key microbial taxa

## Abstract

Vegetation recovery is an important marker of ecosystem health in the mining area. Clarifying the influence of vegetation recovery on the characteristics of soil microbial community and its assembly process can improve our understanding of the ecological resilience and self-maintaining mechanism in the open-pit mining area. For this purpose, we employed MiSeq high-throughput sequencing coupled with null model analysis to determine the composition, molecular ecological network characteristics, key bacterial and fungal clusters, and the assembly mechanism of the soil microbial communities in shrubs (BL), coniferous forest (*CF*), broad-leaved forests (BF), mixed forest (MF), and the control plot (CK, the poplar plantation nearby that had been continuously grown for over 30 a without disturbance). The results showed that the vegetation restoration model had a significant influence on the α-diversity of the microbial community (*p* < 0.05). Compared with CK, Sobs and Shannon index of MF and *CF* have increased by 35.29, 3.50, and 25.18%, 1.05%, respectively, whereas there was no significant difference in the α-diversity of fungal community among different vegetation restoration types, *Actinobacteria*, *Chloroflexi*, *Proteobacteria*, and *Acidobacteria* were the dominant phyla. The diversity of the first two phyla was significantly higher than those of CK. However, the diversity of the last two phyla was dramatically lower than those of CK (*p* < 0.05). *Ascomycota* and *Basidiomycota* were dominant phyla in the fungal community. The abundance and diversity of *Ascomycota* were significantly higher than those of CK, while the abundance and diversity of the latter were considerably lower than those of CK (*p* < 0.05). The stochastic process governed the assembly of the soil microbial community, and the contribution rate to the bacterial community construction of CK, *CF*, BF, and MF was 100.0%. Except for MF, where the soil fungal community assembly was governed by the deterministic process, all other fungal communities were governed by the stochastic process. *Proteobacteria* and *Acidobacteria* are key taxa of the bacterial network, while *Mortierellales*, *Thelebolales*, *Chaetothyriales,* and *Hypocreales* are the key taxa of the fungal network. All these results might provide the theoretical foundation for restoring the fragile ecosystem in the global mining region.

## Introduction

Open-pit mining can cause extremely severe damage to the ecosystem on the earth surface. Soil and vegetation damage is widespread in the open-pit mining area (Wang et al., 2016). The local eco-environment is usually altered, which further affects the biodiversity and functional stability of the ecosystem (Li et al., 2014; Baldrian, 2017). The Loess Plateau, an ecologically vulnerable region, produces about 30% of the world’s total coal. Mining has aggravated surface collapse and water and soil loss, leading to soil depletion and vegetation degradation (Festin et al., 2019; Gastauer et al., 2019). All these ecological phenomena have severely restricted regional sustainable development. Ecological repair and governance are pressing issues at the moment. Vegetation recovery measures the degree of ecological restoration in the ecologically damaged mining areas (Crouzeilles et al., 2016; Zhao et al., 2021). Taking full advantage of the self-recovery ability of the soil–plant system is vital for building a relatively stable ecosystem (Bahram et al., 2020; Yang et al., 2020). The Loess Plateau is featured by low precipitation and poor water-and fertility-retaining abilities, especially in the reclaimed soils of the mining area. Vegetation restoration is a highly challenging task because this region has prevalent “small old trees,” and a persistent drought may cause extensive wilting and death of trees. In addition, soil ecosystem conservation and resilience reconstruction tend to be neglected during the manual repair of some mining areas. As a result, the reconstructed soils are too underdeveloped to satisfy the growth needs of higher plants, such as trees. Given the above, stimulating the natural resilience and promoting the self-repair of reconstructed soils are essential for vegetation restoration in the mining areas (Yang et al., 2018; Liu et al., 2019).

In recent years, there has been growing scholarly attention towards the utilization of microbes in soil-vegetation system restoration. Microbes are the most extensively distributed and active constituents in the earth’s surface ecosystem, driving substance cycling and nutrient conversion (Shi et al., 2016; Baldrian, 2017). Microbes are regulators of and catalysts for belowground ecological processes. Therefore, recognizing the interactions between soil microbial species and the mechanism of mutual feedback between microbes and plants can improve the understanding of the functional diversity and roles of the microbial community (Barberan et al., 2012; Faust and Raes, 2012). Wei et al. found that metabolic activities such as Acetobacter respiration and nitrification in soil were closely related to vegetation types. Sharrar et al. (2020) confirmed that the *Acidobacteria* generally existed in the grassland soils of grass, which had the highest biosynthetic potential capacity. It was also mentioned that the enrichment degree of *Actinobacteria* in the grassland was significantly higher than that in the trees, while the subtaxa with the highest biosynthetic potential were enriched in the hills. Some scholars have already studied the features of soil microbial communities after vegetation restoration in the Loess Plateau (Banerjee et al., 2016, 2019; Deng et al., 2016). They are mainly concerned with the composition of the soil microbial community and its correlations with environmental factors (Banerjee et al., 2016; Xu et al., 2022). However, the assembly mechanism of complex microbial communities is rarely discussed. A null model based on β-diversity indices (βNTI and RCbray) was developed to quantify the process of microbial community assembly (Graham et al., 2017). The model offers a tool to quantitatively elucidate the ecological mechanism of soil microbial co-occurrence under different vegetation restoration models and assess the soil microbial community assembly and succession process (Barberan et al., 2012). Considering the soil microbial community, the deterministic and stochastic processes are two mutually complementary mechanisms that play important roles. The deterministic process is determined by non-biological (e.g., pH value and temperature) and biological factors (e.g., competition among individuals of different species and predator–prey relationship). More specifically, the deterministic process comes into play by differentiating preferences and adaptability of microbes to different habitats. Microbial communities vary with the vegetation restoration model and land use type (Zhang et al., 2011, 2016; Thomson et al., 2015). The natural history and physiology of different vegetation types also vary (Eviner, 2004), resulting in the formation of a unique soil environment and biotic communities (Zhao et al., 2021). By contrast, the stochastic process highlights the roles of dispersal and ecological drift (Zhou and Ning, 2017). The soil microbial community can track the changes in the aboveground plant community (Peay et al., 2013). The influence of specific plant species on the composition of the soil microbial community is closely related to the soil type (Bulgarelli et al., 2012). The soil-vegetation interaction is a highly complex process during the ecological restoration of the mining area; it is not only restricted by local natural environment elements but also influenced by foreign soils and alien plants. These two sources of influence control the resilience of the soil-vegetation system (Zhou and Ning, 2017; Mori et al., 2018). Understanding the soil-vegetation interaction is significant for the natural restoration and health of the mining area ecosystem (Mori et al., 2018).

Microbial network analysis is a valuable tool for studying the complex relationship between the species (Barberan et al., 2012; Faust and Raes, 2012) and identifying the interactions between microbial clusters and the key populations (Deng et al., 2016; Nuccio et al., 2016). Microbial network analysis can help monitor and evaluate key microbial clusters and changes in relevant ecological functions (Banerjee et al., 2016; Shi et al., 2018). One study has shown that deterministic factors have a more significant impact on a larger spatial scale (Shi et al., 2018). So, the question is: Is the microbial community assembly in reconstructed soils in the mining area of the Loess Plateau governed by the deterministic process? Or, is the stochastic process exerting the governing effect? Finding an answer to this question can facilitate understanding ecosystem resilience regulation and ecosystem self-maintaining mechanism reconstruction in the ecologically damaged mining area. In the present study, we focused on the reclaimed forests at the Antaibao Open-Pit Mine dumping site in Pingshuo, Loess Plateau. Forty samples were collected from four vegetation types and one control and examined for structural and functional changes in soil microbial communities under different vegetation restoration models. The purpose was to explore the differentiation in the microbial molecular ecological network, key microbial clusters, and their interactions under different vegetation restoration models. This study tried to uncover the influence of vegetation restoration on the soil microbial community assembly in the mining area and the working mechanism. This study offers new insights into the natural restoration of the ecosystem in the ecologically damaged mining area.

## Materials and methods

### Study area

**Figure 1:**
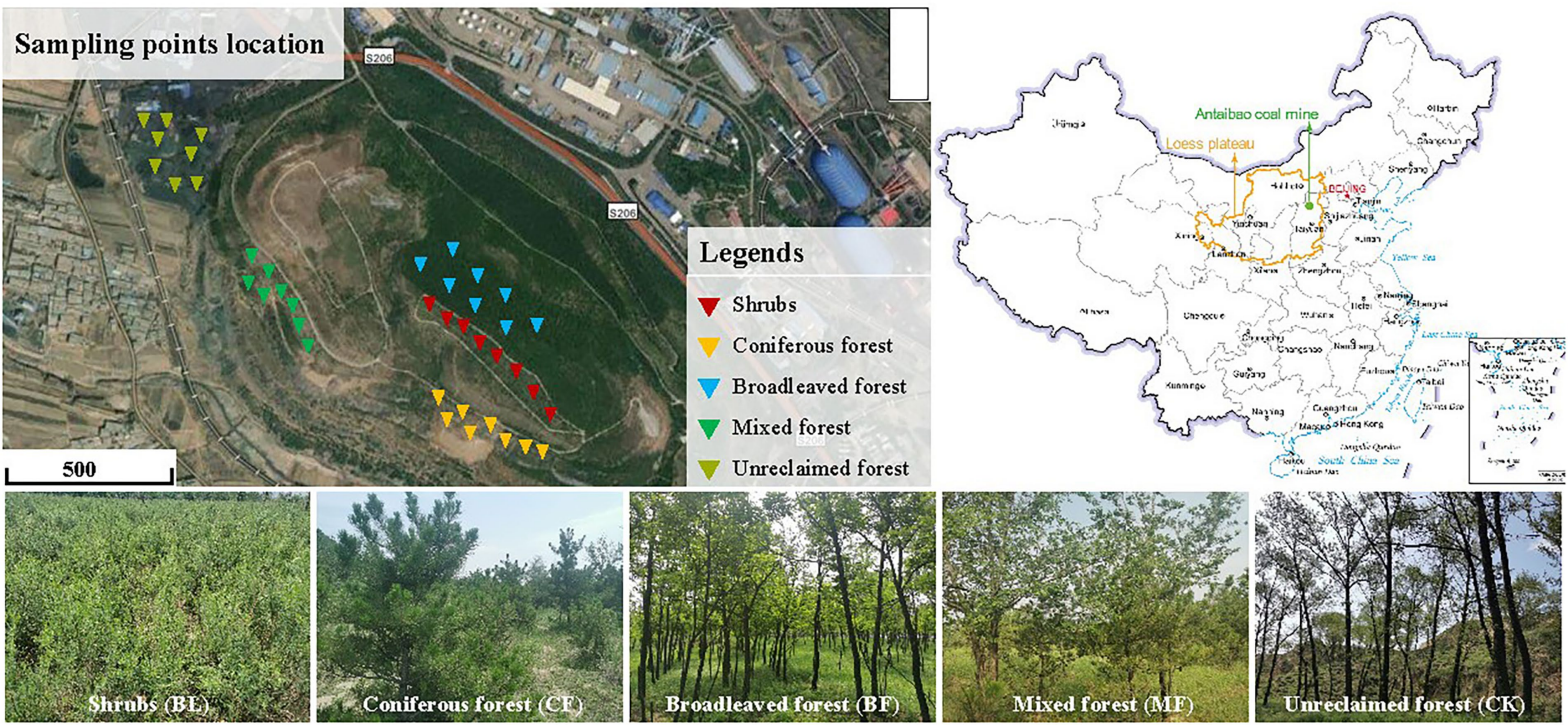
Sampling plots distributed within the study site.

China is the country with the largest coal consumption in the world, accounting for more than 50% of the global coal energy consumption. Most of the theoretical and technical research on mine ecological restoration is concentrated in the middle- and low-altitude areas. The difficulty and effect of restoration depend on natural conditions. The selection of vegetation species in the environment, restrictive factors, material ratio, slope protection mechanism, and restoration effect evaluation will be the key links in the future research on ecological restoration of mine slopes. The south dumping site of the Antaibao open-pit mine is located in Shuozhou, Shanxi Province (112°10′–113°30′E, 39°23′–30°37′N; Figure 1). This mining area has a typical temperate continental semi-arid monsoon climate, with an annual average precipitation of 426.7 mm and an annual average temperature of 5.6°C. However, the precipitation is mostly concentrated from June to September, and the annual average precipitation reaches 2,150 mm. The primary soil type is chestnut soil, with the sand, loam, and clay components accounting for 2.3%, 65.9%, and 31.8%, respectively. The bulk weight of the soil ranged between 1.27 and 1.74 g·cm^−3^, and the organic matter content was found to be low. This dumping site covers an area of 180.5 hm^2^ and has an altitude of 1,465 m. The land reclamation first began in 1992 through layer-by-layer bulldozing and compaction based on height difference, followed by backfilling foreign soils to a depth of 50–60 cm. The reclaimed region is designed as a stepped terrain with an alternation of terraces and side slopes and a relative height of 140 m (Li et al., 2014). The primary plants grown for restoration included: *Pinus tabulaeformis*, *Robinia pseudoacacia*, *Elm*, *Willow*, *Pinus tabulaeformis* + *Elm*, *Caragana*, and *Seabuckthorn*. *Stipa sareptana* and *Elymus dahuricus* were aerially seeded.

## Sample collection and analysis

From July 29 to 30, 2020, we collected soil samples from the shrubs (BL) where *Caragana* species were dominant, coniferous forest (*CF*) comprising *Pinus tabulaeformis* alone, broadleaved forest (BF) comprising *Robinia pseudoacacia* alone, mixed forest (MF) composed of *Pinus tabulaeformis* + *Elm*, and unreclaimed plot (CK). For the collection of each sample, about 200 g of topsoil was dug up from a depth of 0–10 cm at each of the five points chosen randomly. The five parts were combined into a 1,000 g compound sample. Eight duplicates were set up for each vegetation type, and 40 samples were collected in total. The plant roots and gravel were removed on site. The soil samples were mixed adequately, sealed under sterile conditions, and stored in the car fridge at −20°C. Parts of the fresh soil samples were directly refrigerated in the foam boxes, sealed, and mailed to Majorbio Bio-pharm Technology (Shanghai) for sequencing.

### Bioinformatics analysis

Total RNA was extracted from the soil samples according to the instruction provided with the E.Z.N.A.^®^ soil kit (Omega Bio-Tek, Norcross, GA, United States). DNA concentration and purity were determined using a NanoDrop 2000 spectrophotometer. The DNA quality was assessed by 1% agarose gel electrophoresis. For the soil bacteria, the V3-V4 region of 16S rDNA was amplified by polymerase chain reaction (PCR) using primers 338F (5′-ACTCCTACGGGAGGCAGCAG-3′; Xu et al., 2016) and 806R (5′-GGACTACHVGGGTWTCTAAT-3′; Adams et al., 2013). For the soil fungi, the ITS1-ITS2 region of the rRNA gene was amplified by PCR using primers ITS1F (5′-CTTGGTCATTTAGAGGAAGTAA-3′) and ITS2R (5′-GCTGCGTTCTTCATCGATGC-3′). The following were the steps of PCR: Predenaturation at 95°C for 3 min, 27 cycles of denaturation at 95°C for 30 s, annealing at 55°C for 30 s, extension at 72°C for 45 s, and a final extension at 72°C for 10 min (PCR instrument: ABI GeneAmp^®^ 9700). A 20 μl reaction system consisted of 4 μl *5FastPfu buffer, 2 μl 2.5 mM dNTPs, 0.8 μl primer (5 μM), 0.4 μl FastPfu polymerase, and 10 ng DNA template. The PCR products were resolved by 2% agarose gel electrophoresis and purified using the AxyPrep DNA Gel Extraction Kit (Axygen Biosciences, Union City, CA, United States). The proteins were eluted with Tris–HCl and assessed for their quality by 2% agarose gel electrophoresis. Quantification was done using QuantiFluor™ -ST (Promega, United States). The PE 2*300 library was constructed using the purified amplification products on the Illumina MiSeq platform (Illumina, San Diego, United States) following the standard operating procedure. Sequencing was done on the Illumina MiSeq (PE300) sequencing platform (Majorbio, Shanghai).

After performing quality control for the original sequencing data using Trimmomatic, the reads were merged using the FLASH software. However, after the quality control and merging of the reads, PCR and sequencing errors might still exist in the optimized sequences; hence, the optimized data were further processed using the sequencing denoising algorithm DADA2 or Deblur to eliminate these errors as much as possible and obtain the amplicon sequence variants (ASVs). The sequence and abundance information, as represented by ASVs, were used for the subsequent taxonomic analysis of the species, microbial community diversity analysis, and species composition analysis. The original 16S rRNA sequence data of bacteria and fungi are already stored in the NCBI database (accession numbers PRJNA725677 and PRJNA725679, respectively).

### Network analysis

The molecular ecological network analysis (MENA) was constructed using the Network Analyses Pipeline.^1^ The ASVs were subjected to the 1og10 transformation. The correlation matrix was computed and converted into a similarity matrix based on Spearman’s correlation coefficient (Deng et al., 2016). According to the random matrix theory, the optimal similarity threshold was selected from the reciprocal adjacency matrix of the similarity matrix. The intensity of connection between each pair of nodes was coded using the adjacency matrix. The ecological communities were predicted by analyzing the nearest neighbor spacing distribution of eigenvalues (Zhou et al., 2010; Deng et al., 2012). The network typology properties were described by calculating the average clustering coefficient (avgCC), average path distance (APD), graph density (GD), and modularity (M). The role of each node in the typology was determined based on two attributes, intramodular connectivity (*Zi*) and intermodular connectivity (*Pi*; Guimera and Amaral, 2005). Using the thresholds for different levels of *Zi* and *Pi* as recommended by MENA (Zhou et al., 2010; Derocles et al., 2018), all species were divided into four topological roles: peripherals (*Zi* < 2.5, *Pi* < 0.62), module hubs (*Zi* > 2.5, *Pi* < 0.62), connectors (*Zi* < 2.5, *Pi* > 0.62), and network hubs (*Zi* > 2.5, *Pi* > 0.62). The network was visually analyzed using the Gephi software.

### Data processing and statistical analysis

The influence of the vegetation restoration model on soil microbial diversity was assessed by one-way analysis of variance (ANOVA) and the least significant difference (LSD) test. The microbial alpha-diversity analysis, PCoA, and analysis of species differences were conducted on the Majorbio Cloud platform.^2^ Based on the Bray-Curtis distance, PCoA was employed to assess the overall structural differences of the soil microbial community. The difference in relative abundance on the order level was assessed using the Kruskal–Wallis H test. The null model was used to analyze the ecological process of the microbial communities and the microbial community assembly on the Galaxy/DengLab platform^3^ (Stegen et al., 2013). Each ASV on the dataset was analyzed through Reconstruction of Unobserved States (PICRUSt2) to predict the metabolic status of the bacterial community.^4^ Based on KO (KEGG orthologs) levels 1 and 2, the relative abundance of functional traits and functional genes were analyzed to determine the differences among the treatment. We first calculated the parameters, such as the β-nearest taxon index (βNTI) and the Raup-Crick matrix (RCbray). When |βNTI| > 2, the community assembly was a deterministic process, and when |βNTI| < 2, the community assembly was a primarily stochastic process. Meanwhile, RCbray > 0.95, RCbray < −0.95, and |RCbray| < 0.95 corresponded to dispersal limitation, homogenizing dispersal, and drift, respectively (Zhou and Ning, 2017).

## Results

### Structural characteristics of the soil microbial communities under different vegetation restoration models

As shown in Table 1, the α-diversity indices of bacterial communities under different vegetation restoration models varied significantly (*p* < 0.05). Compared with CK, the Sob index, Shannon index, and phylogenetic diversity index (PD) increased by 35.29%, 3.50%, and 25.29%, respectively, for the bacterial community in MF. However, the vegetation type had no significant influence on the Sobs and Shannon indices of the fungal community. Compared with CK, PD of the fungal community in BF decreased by 29.5%.

**Table 1:** Alpha (α)-diversity indices of bacterial and fungal communities in soil under different vegetation restoration models.

Treat	Bacterial			Fungi		
	Sobs	Shannon index	Phylogenetic diversity	Sobs	Shannon index	Phylogenetic diversity
CK	1130.38 ± 104.05b	6.56 ± 0.08c	122.51 ± 8.12c	265.25 ± 38.78a	3.13 ± 0.46a	79.01 ± 9.83a
BL	1221.75 ± 53.78b	6.53 ± 0.06c	130.68 ± 3.96bc	240.75 ± 18.23a	3.44 ± 0.22a	71.56 ± 6.54ab
*CF*	1415.00 ± 38.00a	6.72 ± 0.03ab	143.96 ± 3.36ab	227.38 ± 16.34a	3.57 ± 0.15a	67.41 ± 4.79ab
BF	1423.50 ± 59.45a	6.64 ± 0.04bc	150.98 ± 5.10a	205.25 ± 21.62a	3.17 ± 0.22a	55.70 ± 5.64b
MF	1529.25 ± 50.33a	6.79 ± 0.03a	153.49 ± 2.39a	256.13 ± 16.88a	3.33 ± 0.27a	65.44 ± 3.23ab

Different alphabets represent the significant difference in one-way analysis of variance and the least significant difference test at the 5% level across the treatments (*p* < 0.05). The observations were expressed as mean ± standard error (*n* = 8), and the same below.

**Figure 2:**
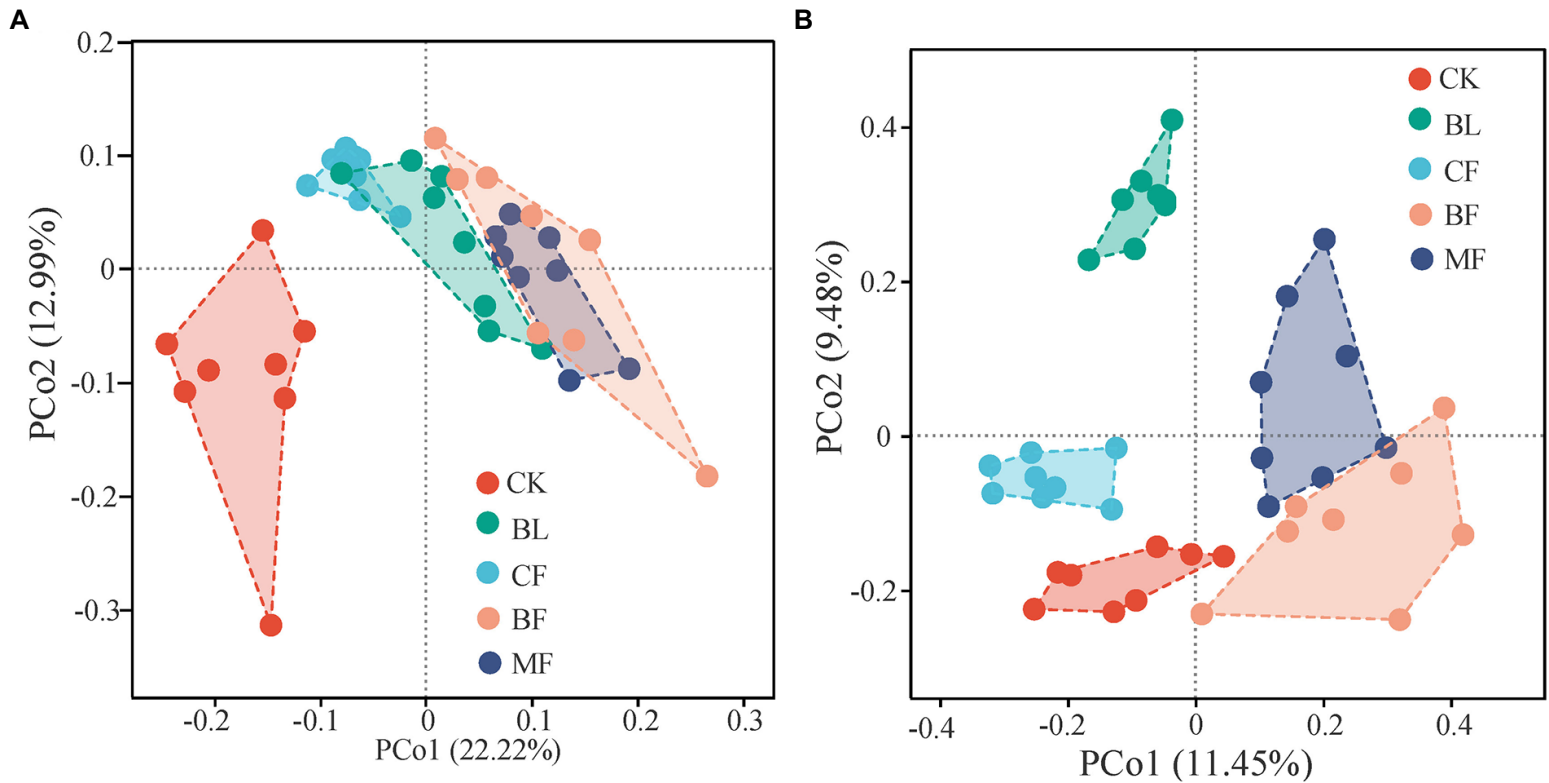
PCoA of bacterial **(A)** and fungal **(B)** β-diversity under different vegetation restoration models.

As shown in Figure 2, the vegetation restoration model had a significant influence on the microbial community structure. Axes 1 and 2 explained 22.2% and 12.9%, respectively, of the β-diversity variation in the bacterial community on the operational taxonomic units (out) level. The sample points of CK were segregated from those of BF and MF along axis 1; the sample points of BF and MF were closer to each other than to others (Figure 2A). The first two principal coordinates explained 20.9% of the β-diversity variation in the fungal community on the OTU level. Sample points of *CF* were significantly segregated from those of BF and MF along axis 1; the sample points of CK were segregated from those of BL along axis 2 (Figure 2B). These results explained the dissimilarity and similarity in microbial community diversity across the sample points under different treatments.

### Structural and functional changes in the soil microbial communities under different vegetation restoration models

**Figure 3:**
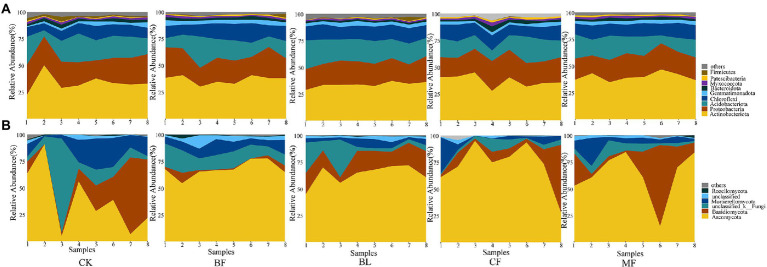
Relative abundance of soil bacterial **(A)** and fungal **(B)** communities at the phylum level.

**Figure 4:**
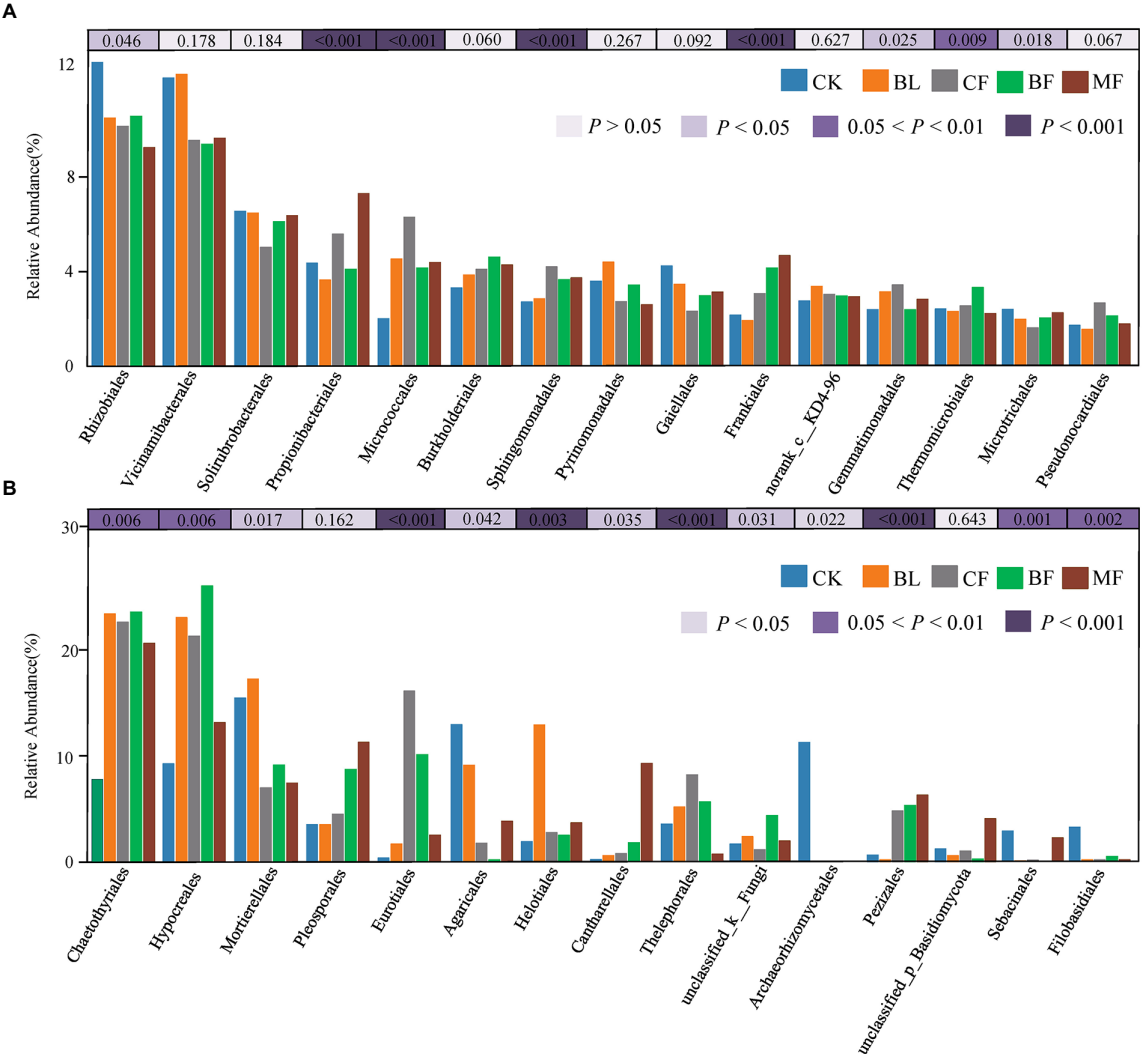
Relative abundances of top 15 bacterial **(A)** and fungal **(B)** order that showed significant differences among rehabilitation modes. Kruskal–Wallis H test was used to evaluate the significance of differences between the indicated groups. ^*^p < 0.05, ^**^p < 0.01, ^***^p < 0.001.

Among the soil bacteria, phyla Actinobacteria accounted for 33%–40%, Proteobacteria accounted for 21%–24%, *Acidobacteria* accounted for 16%–21%, and *Chloroflexi* accounted for 10%–13% of the total relative dominance (Figure 3A). Among the soil fungi, phyla *Ascomycota* accounted for 28%–72%, and *Basidiomycota* accounted for 3%–29% of the dominance (Figure 3B). Compared with CK, the relative abundance of *Ascomycota* increased significantly under any vegetation restoration model (*p* < 0.05), while the relative abundance of *Basidiomycota* was the lowest in BF. Among different treatments, *Rhizobiales* accounted for 9%–13%, *Propionibacteriales* accounted for 4%–7%, and *Micrococcales* accounted for 2%–6% of all bacteria. There was a significant difference in the relative abundance of these bacteria under different vegetation restoration models (Figure 4A). Among the fungi, *Chaetothyriales* accounted for 7%–31%, *Hypocreales* accounted for 9%–26%, and *Mortierellales* accounted for 7%–17% of the relative abundance. There was a significant difference in the relative abundance of these fungi under different vegetation restoration models (Figure 4B).

**Figure 5:**
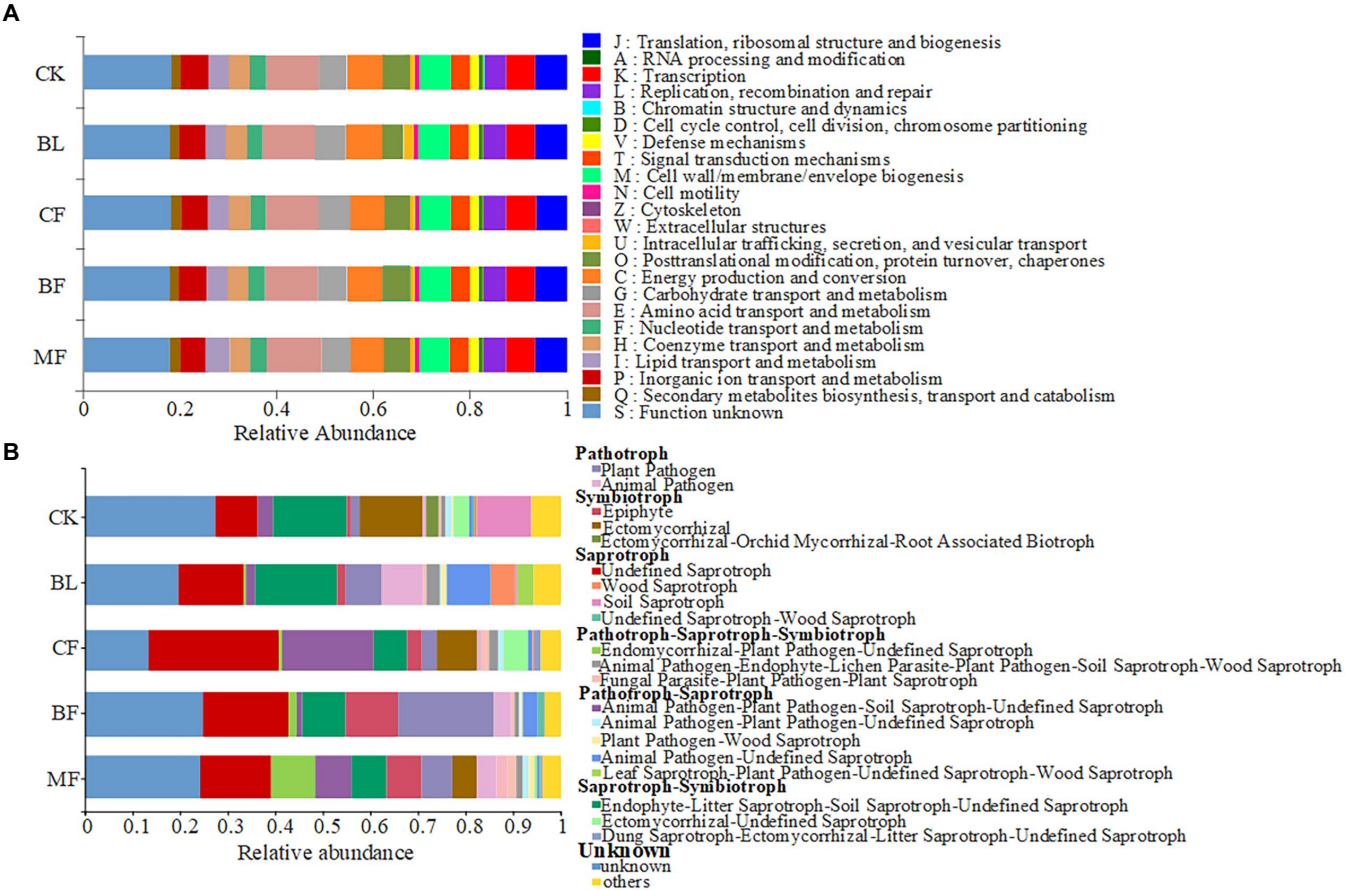
Variations in the composition of bacterial **(A)** and fungal **(B)** functional groups inferred by the PICRUSt2 and FUN Guild, respectively.

Clusters of Orthologous Groups (COG) analysis was conducted using PICRUSt2. The soil bacterial communities under different vegetation restoration models shared similar functional features (Figure 5A). Twenty-two bacterial functions and metabolic pathways were identified, including those for energy production and conversion, amino acid transport and metabolism, nucleotide transport and metabolism, carbohydrate transport and metabolism, and coenzyme transport and metabolism. However, the FUNGuild-based functional prediction indicated significant differences in the functions of soil fungal communities under different vegetation restoration models (Figure 5B). Syntrophic mycorrhizae were mainly found in CK, *CF*, and MF samples; pathographic plant pathogens were mainly found in BF samples. Though the abundance distribution was uneven, undefined saprophytic fungi were widespread across the treatments.

### Characteristics of the molecular ecological network of soil microbial communities under different vegetation restoration models

**Figure 6:**
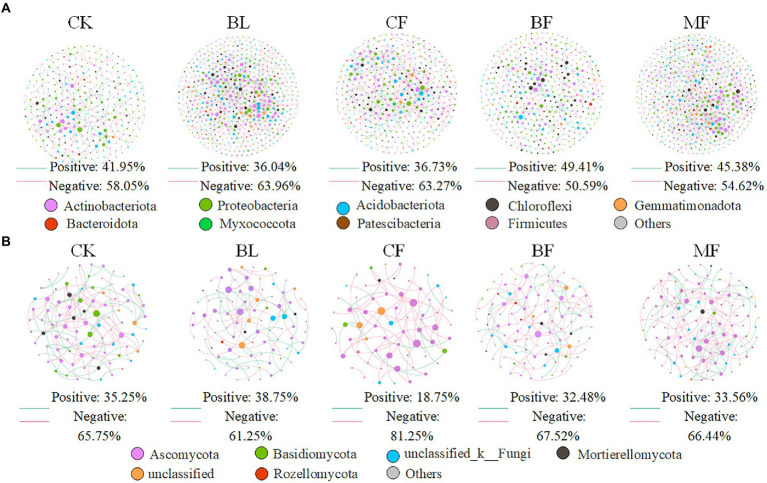
Co-occurrence network of bacteria **(A)** and fungi **(B)** under differences among rehabilitation types. Each node represents individual amplicon sequence variants (ASVs). The size of each node is proportional to the number of connections (that is, degrees). The nodes are colored at the phylum level, and an edge represents a Spearman correlation with a correlation coefficient of >0.6, a green edge represents a positive interaction, and a red edge represents a negative interaction.

We further constructed the soil bacterial and fungal co-occurrence network under different vegetation restoration models (Figure 6). In all bacterial networks, the identifiable dominant ASVs were primarily assigned to *Actinobacteria*, *Proteobacteria*, and *Acidobacteria* (Figure 6A). Compared with CK, the soil bacterial networks under different vegetation restoration models were more complex and had more nodes and edges. The average path distance and the modularity were smaller, while the average connectivity and clustering coefficient was higher. Besides, most nodes in the soil bacterial networks under different vegetation restoration models were peripherals, indicating closer connections between bacterial species. In all fungal networks, the identifiable dominant ASV was assigned to *Ascomycota* (Figure 6B). Compared with CK, the fungal network of MF had more nodes and edges. In fungal communities, the interspecies relationship tended to be co-excluded (negative relationship, red line).

**Figure 7:**
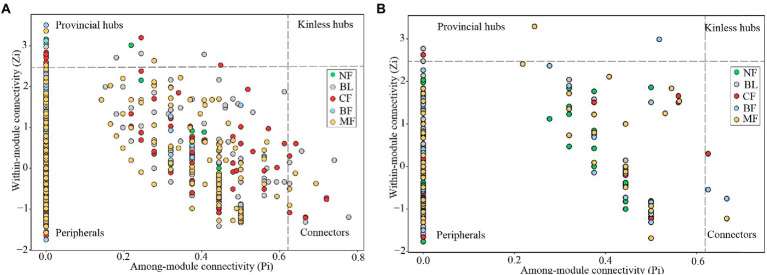
Z-P plot showing the distribution of amplicon sequence variants (ASVs) based on their topological roles. Each color represents a kind of vegetation type. The topological role of each ASV was determined according to the scatter plot of within module connectivity (*Zi*) and among-module connectivity (*Pi*). **(A)** Z-P plot of bacterial community MENs; **(B)** Z-P plot of fungal community molecular ecological networks (MENs).

Vegetation restoration had a significantly differential impact on module hubs in the bacterial networks. *Chloroflexi* only appeared in the bacterial networks of BL and *CF*, *Actinobacteria* and *Proteobacteria* appeared in the bacterial networks of CK, BL, and *CF*; *Methylomirabilota* and *Armatimonadota* appeared in the bacterial networks of CK and *CF*; *Proteobacteria* was absent only in the bacterial network from BF; and *Acidobacteria* was the module hub in all networks. Besides, *Burkholderiales*, *Sphingomonadales*, and *Rhizobiales*, orders *Proteobacteria*, *Vicinamibacterales*, and *Acidobacteria*, were also module hubs. The bacterial networks from BL, *CF*, and MF had connected nodes, but those from CK and BF did not (Figure 7A). Compared with CK, the fungal network of MF had more nodes and edges. ASV204 assigned to Ascomycota was the module hub of the fungal network from BF. ASV83 assigned to *Mortierellomycota* was the module hub of the fungal network from MF. The fungal networks from *CF*, BF, and MF had connected nodes (Figure 7B), all of which were assigned to the Ascomycota. However, these nodes could be assigned to different orders, including *Thelebolales*, *Chaetothyriales*, and *Hypocreales*. There were no connected nodes in the fungal network of CK and BL (Figure 7B; Table 2).

**Table 2:** Soil bacterial and fungal community network parameters in different vegetation restoration types.

	Network parameters	NF	BL	*CF*	BF	MF
Bacteria	avgCC	0.153	0.178	0.225	0.179	0.209
	APD	11.57	6.987	6.841	6.979	7.508
	GD	0.007	0.005	0.007	0.005	0.007
	M	0.905	0.804	0.812	0.934	0.777
Fungi	avgCC	0.238	0.067	0.121	0.134	0.153
	APD	6.068	3.82	3.92	6.085	5.447
	GD	0.03	0.02	0.031	0.022	0.025
	M	0.747	0.856	0.755	0.768	0.742

avgCC, clustering coefficient; APD, average path distance; GD, graph density; M, modularity.

### Assembly process of soil microbial community for different vegetation restoration models

**Figure 8:**
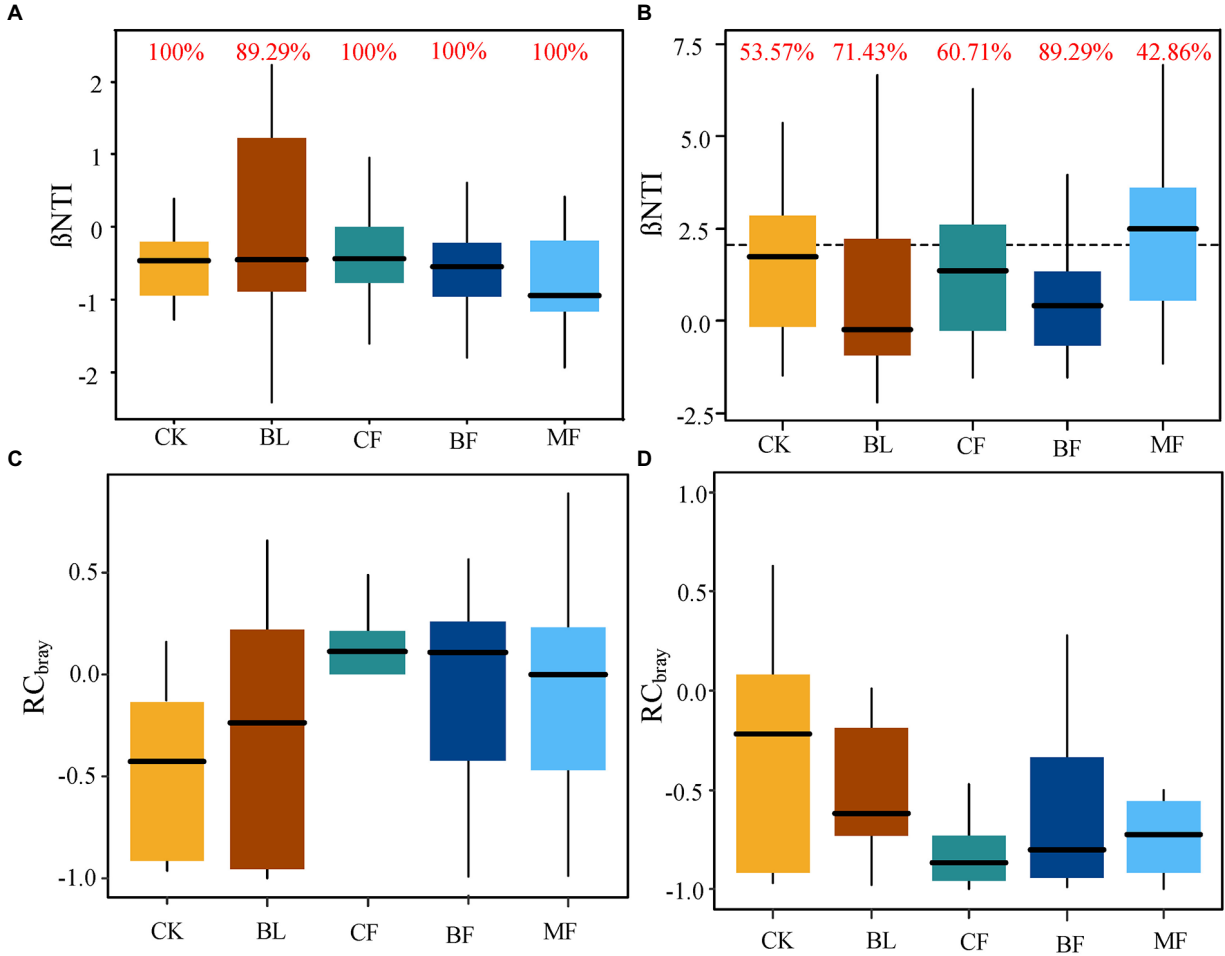
**(A,C)** were the β-nearest taxon index (βNTI) and Bray-Curtis-based Raup-Crick (RCbray) box plots of bacterial communities under different vegetation reconstruction models. **(B,D)** present βNTI and RCbray box plots of fungal communities under different vegetation reconstruction models. The red numbers indicate the contribution rate of random processes to the construction of microbial communities.

The calculated values of βNTI and RCBray were further compared. For soil bacteria, |βNTI| < 2 (Figure 8A) indicated that the phylogenetic differences were controlled by the stochastic process. The contribution rate to bacterial community assembly was 100.0% for CK, *CF*, BF, and MF, respectively. For all bacterial communities, |RCbray| < 0.95 indicated that the bacterial community assembly was not governed, but weak selection, weak dispersal, speciation, and drift acted jointly (Figure 8C). For soil fungal communities from CK, BL, *CF*, and BF, |βNTI| < 2 indicated that the fungal community assembly was governed by the stochastic process (Figure 8B). Meanwhile, |RCbray| < 0.95 for fungal communities indicated that the community was assembled under the joint action of weak selection, weak dispersal, speciation, and drift, while the community assembly was not governed (Figure 8D). However, βNTI > 2 for the fungal community from MF indicated that the deterministic process governed the soil fungal community assembly in MF. Meanwhile, the contribution rate of the stochastic process was 42.86% (Figure 8B). As shown in Figure 8, irrespective of the vegetation restoration model, the governing effect exerted by the stochastic process over the microbial community assembly far surpassed that over the fungal community.

## Discussion

### Influence of vegetation restoration on the diversity and functions of the microbial communities

Microbes are indispensable in regulating ecological processes and functions. Vegetation restoration in the mining area can relieve soil environmental stress and promote the development of microbial flora (Delgado-Baquerizo et al., 2018). We found that the abundance and diversity of microbial communities from *CF* and MF were significantly higher than those from CK, indicating that *CF* and MF were more conducive to microbial community diversity, consistent with the previous studies (Yang et al., 2020). Under different vegetation restoration models, the dominant soil bacterial phyla were consistently *Actinobacteria*, *Proteobacteria*, *Acidobacteria*, and *Chloroflexi*, congruent with those reported in other mining areas of the Loess Plateau (Guo et al., 2020; Jia et al., 2020; Shi et al., 2020). However, the dominant bacterial flora did not vary significantly on the phylum level across the vegetation restoration models, but the difference was significant at the level of order (Figure 4). One dominant phylum in the study area was oligotrophic *Acidobacteria*, indicating soil depletion in the mining area of the Loess Plateau (Thomson et al., 2015). *Acidobacteria* exist in various environments, accounting for about 20% of the total bacteria, and some even reach more than 50%. The bacteria of *Acidobacteria* and *Proteobacteria* in the soil are equivalent in number, and they play an important role in the process of soil material circulation and ecological environment construction (Lee et al., 2008). Naether et al. (2012) showed that there were significant differences in the community structure of *Acidobacteria* between forest and grassland soil. Soil pH, organic carbon, total nitrogen, C/N ratio, phosphorus, nitrate nitrogen, ammonium nitrogen, soil moisture, soil temperature, and soil respiration, were all confirmed to have effects on the composition of the *Acidobacteria* community structure. Firere et al. demonstrated that the relative abundance of *Acidobacteria* in forest soil was significantly positively correlated with mean precipitation and soil organic carbon, while negatively correlated with soil pH value. Different vegetation types could lead to different soil physicochemical environment. Another dominant phylum was eutrophic *Proteobacteria*, indicating the abundance of carbon-containing organic pollutants in the soil of the mining area, which supply unstable carbon substrates for such *Proteobacteria* (Kurm et al., 2017). PICRUSt2-based functional prediction showed that the vegetation restoration type had no significant impact on the functions of the microbial community, which agreed with those reported in other mining areas of the Loess Plateau (Jia et al., 2020; Zhao et al., 2021).

The dominant soil fungal phyla in the study area were *Ascomycota* and *Basidiomycota*. The relative abundance of *Ascomycota* was higher, and most species of this phylum were saprophytic bacteria, insensitive to environmental stress (Ventorino et al., 2016). The relative abundance of *Basidiomycota* was significantly higher in MF, while that in BF was lower. *Basidiomycota* are important decomposers. As widespread ectomycorrhizal fungi (Zhong et al., 2020), they can promote soil nutrient recycling in the mining area. Given the above, the soil in BF can be suitably inoculated with *Basidiomycota* species. Besides, the FUNGuild-based functional prediction showed that the fungal functions varied significantly across the vegetation restoration models. Vegetation restoration had a much greater impact on the abundance of functional fungal clusters than on functional bacterial clusters. Similar reports have been published previously (Peay et al., 2013; Bahram et al., 2020). The reason is mostly that plant pathogenic fungi and ectomycorrhizal fungi live in symbiosis with the root systems and thus have closer connections to vegetation than bacteria (Guo et al., 2020).

### Influence of vegetation restoration on the molecular ecological network of the soil microbial community

Compared with CK, the bacterial networks in BL, *CF*, and MF had more nodes and edges and hence a higher complexity. The bacterial network in BF had a significantly higher average clustering coefficient and modularity but a lower average path distance. This indicated that the bacterial network in MF was a self-contained “small-world” module. The bacterial clusters within the module were closely interconnected and shared many similarities (Ling et al., 2016). Generally speaking, bacterial clusters serving as module hubs and connectors in the topology structure were key clusters (Derocles et al., 2018), which played a crucial role in system stability. Module hubs in soil bacterial networks under different vegetation restoration models were all *Proteobacteria* and *Acidobacteria*, which were closely related to the adaptation of eutrophic *Proteobacteria* to the complex carbon environment of the mining area (Kurm et al., 2017). *Acidobacteria* are particularly adapted to the mining area (Banerjee et al., 2016). We found that *Burkholderiales*, *Sphingomonadales*, *Rhizobiales*, and *Vicinamibacterales* were module hubs of the bacterial networks. *Ascomycetes* are saprophytic fungi, and decomposing plant residues and degrading organic matter are their main functions. The main function of *Basidiomycetes* is to decompose lignified vegetation debris. The rich contents of CK and BF organic matter may affect the content of *Ascomycetes*. The high lignin content of shrubs and trees, coupled with moist soil, could provide proper living conditions for the growth of *Basidiomycetes*. The results of this study showed that saprophytic fungi accounted for the largest proportion of each fungal function, while MF and BF could improve the enrichment of soil saprophytic fungi. Previous studies have shown that symbiotic trophic fungi may play an important role in soil health and nutrient cycling (Jing et al., 2021). It is worth noting that the symbiotic trophic fungi of *CF* and MF are the most abundant, and they have been reported to have the ability of reducing the risk of soil pests and diseases and improving soil quality. Attempts can be made in the future to isolate and purify the key bacterial species along with their amplification and inoculation to facilitate soil-vegetation restoration of the mining areas.

*Ascomycota* were observed to be module hubs in all soil fungal networks under different vegetation restoration models, playing key roles in the fungal network. Co-excluded negative relationships were predominant among clusters in the fungal networks, accounting for 61%–81% of potential interactions. The negative relationship was attributed to competition and homogenization (Faust and Raes, 2012), which enhanced the stability of the ecological network under the mining disturbance (de Vries et al., 2018). Compared with CK, vegetation restoration reduced the average clustering coefficients of the fungal networks. The external disturbance would not influence the entire fungal community within a short period, indicating increased resistance. *Mortierellales*, *Thelebolales*, *Chaetothyriales*, and *Hypocreales* were key clusters in the fungal networks. Species of these orders were isolated and purified and would be amplified and inoculated to the root systems of plants used for restoration. In the future, we will step up the exploration into relevant functional microbes to enhance the understanding of the mechanism of ecological restoration in the mining area.

### Influence of vegetation restoration on the process of soil microbial community assembly

Deterministic and stochastic processes jointly regulated microbial community assembly (Zhou and Ning, 2017; Mori et al., 2018), though the relative importance of each varied across the environments (Tian et al., 2017). In the current study, |βNTI| < 2 for all soil bacterial communities indicated that the stochastic process governed bacterial community assembly. Moreover, |RCbray| < 0.95 for the soil bacterial communities indicated that the assembly process was not governed (Zhou and Ning, 2017). Vegetation restoration in the mining area only constituted a local disturbance, which was quite different from the influence of large-scale spatial processes such as temperature and precipitation. Our result also demonstrates that the stochastic process usually occurs in small-scale spaces with environmental changes (Shi et al., 2018). Although the stochastic process is hard to predict (O’Malley, 2008), the stochastic factors influencing microbial community assembly are more or less predictable. Therefore, we can predict the probable process of microbial community assembly by exploring when stochastic factors take effect and when they do not (Fukami, 2015). Except for MF, |βNTI| < 2 for the fungal communities in CK, BL, BF, and *CF* indicated the governing role played by the stochastic process in fungal community assembly. Meanwhile, |RCbray| < 0.95 for all fungal communities indicated that the fungal community assembly was jointly governed by weak selection, weak dispersal, speciation, and drift. In addition to the lesser impact of selection, mutual cancellation or contrasting options might also exist, leading to a stochastic development model of soil microbial flora in the mining area (Zhou and Ning, 2017). For the fungal community in MF, βNTI>2 indicated that the deterministic process governed fungal community assembly in MF, which might be further attributed to heterogeneous selection (Zhou and Ning, 2017). The microbial community assembly process can become more predictable if we explore the temporal and conditional factors under which the microbial communities are more sensitive to the stochastic process (Fukami, 2015). On this basis, we can appropriately manipulate microbial community assembly to achieve the desired ecosystem functions, thereby assisting with ecological restoration and biodiversity protection and management in mining areas (Chase, 2010).

Ecosystem monitoring, as the key construction project, is always an important pathway to deal with environmental changes, especially in mining areas. This study has shown that different vegetation restoration types have significantly changed the soil microbial molecular ecological networks in the mining area. In relatively healthy mining areas, the microbial molecular ecological network was more complex and closely connected, and the interaction relationship is also more complicated. However, the links between soil microbial molecular ecological networks in the damaged mining areas tended to be single, while the connections between various nodes were less, as well as the network was simplified. Then this obvious regularity could be used to quantitatively and visually express the microscopic changes of the environment in the mining area, as well as monitor and evaluate the ecological succession law of the damaged mining area. Through comparing the changes of soil microbial molecular ecological network interaction between healthy mining areas and damaged mining areas, land reclamation and ecological restoration strategies could be reasonably formulated, while soil reconstruction work could be guided to a reasonable evolution direction. At the same time, the introduction of soil microbial molecular ecological network monitoring system will provide new ideas for improving the evaluation of mining ecosystem.

## Conclusion

Studies have shown that different vegetation restoration types have significantly different effects on soil microbial community diversity. Compared with CK, BF and MF significantly changed the soil bacterial community structure, but did not change the bacterial community composition. BL, *CF,* and MF significantly altered the fungal community structure and increased the relative abundance of *Ascomycota* and decreased the relative abundance of *Basidiomycota*. *Ascomycota* and *Basidiomycota* are the dominant phyla in the fungal community. Different vegetation restoration types shaped different fungal functional groups, but there was no significant difference in bacterial functions. Vegetation restoration increased soil microbial community network and complexity. Compared with CK, the BL, *CF*, and MF bacterial networks were larger and more complex, and the MF fungal network was more complex and stable. *Acidobacteria* and *Proteobacteria* were the hubs and key taxa of the bacterial network module, while *Ascomycota* and *Mortierella* played key roles in BF and MF fungal community networks, respectively. The soil bacterial assembly process was dominated by stochastic processes and was hardly affected by the type of vegetation restoration. However, the MF fungal community assembly is dominated by deterministic processes, and the stochastic process controls the bacterial community assembly process much more than the fungal community. Clarifying the time and conditions when microbial communities are more sensitive to random processes will help to manipulate community assembly and achieve required ecological functions, thereby scientifically guiding ecological restoration, biodiversity conservation, and management in mining areas.

## Data availability statement

The datasets presented in this study can be found in online repositories. The names of the repository/repositories and accession number(s) can be found in the article/supplementary material.

## Author contributions

YuC, YZ, and JM collected the samples. YaC, YZ, and YY performed the experiments. YuC performed the data analyses and wrote the manuscript. FC, JM, and YZ helped perform the analysis with constructive discussions. FC performed the supervision, project administration, and funding acquisition. All authors contributed to the article and approved the submitted version.

## Funding

This work was supported by the National Natural Science Foundation of China (no. 51974313) and the key project of Jiangsu Key Laboratory of Coal-based Greenhouse Gas Control and Utilization (2020ZDZZ03). We gratefully acknowledge these programs for financial support.

## Conflict of interest

The authors declare that the research was conducted in the absence of any commercial or financial relationships that could be construed as a potential conflict of interest.

## Publisher’s note

All claims expressed in this article are solely those of the authors and do not necessarily represent those of their affiliated organizations, or those of the publisher, the editors and the reviewers. Any product that may be evaluated in this article, or claim that may be made by its manufacturer, is not guaranteed or endorsed by the publisher.

## Footnotes

^1^ http://ieg4.rccc.ou.edu/mena

^2^ www.majorbio.com

^3^ http://mem.rcees.ac.cn:8080/

^4^ https://cloud.majorbio.com/
